# Return to Work Following Anterior Lumbar Interbody Fusion with Percutaneous Posterior Pedicle Fixation: A Retrospective Analysis from Two Academic Centers in Germany

**DOI:** 10.3390/jcm13185636

**Published:** 2024-09-23

**Authors:** Bedjan Behmanesh, Helen Wempe, Fatma Kilinc, Daniel Dubinski, Sae-Yeon Won, Marcus Czabanka, Matthias Setzer, Patrick Schuss, Matthias Schneider, Thomas Freiman, Florian Gessler

**Affiliations:** 1 Department of Neurosurgery, University Medicine of Rostock, Schillingallee 35, 18057 Rostock, Germany; helen.wempe@med.uni-rostock.de (H.W.); daniel.dubinski@med.uni-rostock.de (D.D.); sae-yeon.won@med.uni-rostock.de (S.-Y.W.); thomas.freiman@med.uni-rostock.de (T.F.); florian.gessler@med.uni-rostock.de (F.G.); 2 Department of Neurosurgery, Goethe University Hospital, 60528 Frankfurt am Main, Germany; fatmakilinc@hotmail.de (F.K.); marcus.czabanka@med.uni-frankfurt.de (M.C.); matthias.setzer@med.uni-frankfurt.de (M.S.); 3 Department of Neurosurgery, Unfallkrankenhaus, 12683 Berlin, Germany; patrick.schuss@ukb.de; 4 Department of Neurosurgery, University Hospital Bonn, 53127 Bonn, Germany; matthias.schneider@ukbonn.de

**Keywords:** anterior lumbar interbody fusion, return to work, surgery

## Abstract

**Objective:** Return to work after spinal surgery is a crucial factor in the recovery process. It can contribute not only to physical rehabilitation but also to psychological well-being. This study aims to evaluate the rate of return to work following elective lumbar spine surgery and identify predictors that predict failure of return to work. **Methods:** Adult patients who underwent anterior lumbar interbody fusion at two medical centers were retrospectively identified. A standardized telephone interview was conducted for the final analysis to assess the clinical outcomes of these patients. **Results:** Out of a total of 159 patients, 104 were of working age at the time of the elective surgery. Data were missing for 35 patients, who were thus excluded from the analysis. All patients had a minimum follow-up period of one year. After surgery, 75% of the patients returned to work within a median time of 3 months. Quality of life, back pain, leg pain, and ODI scores, as well as self-reported satisfaction, were significantly better in patients who returned to work (*p* < 0.05). Tobacco use and previous musculoskeletal surgery were significant predictive factors of failure to return to work. None of the patients who were unemployed prior to surgery returned to work. **Conclusions:** Our study reveals that 75% of patients returned to work within three months after surgery. The most significant predictor of failure to return to work is being unemployed before surgery. Additionally, preoperative education about postoperative behavior and physical activity could potentially increase the rate.

## 1. Introduction

Low back pain (LBP) is increasingly recognized as a significant public health issue, with an estimated global lifetime prevalence of up to 80% [1]. It is currently the leading cause of disability worldwide, associated with significant work absenteeism, contributing to a substantial socioeconomic burden and loss of productivity [2]. Among adults under 45, LBP ranks as the second most common reason for physician visits, following the common cold [3]. It is the third leading cause of surgical procedures and the fifth most frequent cause of hospital admissions [3]. The treatment and rehabilitation of patients with LBP pose significant social and economic challenges.

There has been a significant increase in spinal surgeries performed, particularly spinal fusion operations. The rate of spinal fusions has increased more rapidly than many other inpatient procedures. Interbody fusions are the most frequently performed spinal surgeries in the United States, with over 352,000 procedures carried out annually [4,5]. Multiple fusion techniques are available, including anterior lumbar interbody fusion (ALIF), posterior lumbar interbody fusion (PLIF), transforaminal lumbar interbody fusion (TLIF), and posterolateral lumbar fusion (PLF). However, there is no consensus on which technique is most effective for patients with LBP. ALIF offers several advantages compared to posterior fusion, including the complete removal of the disc, which helps restore disc height and lumbar lordosis. It also allows for both direct and indirect neural decompression, accommodates a larger area for graft material, facilitates the insertion of a cage and graft, and reduces the risk of damage to the erector spinae muscles and posterior ligamentous structures. The disadvantages include the potential for injury to blood vessels, internal organs, the parasympathetic and sympathetic nerves, as well as the possibility of muscle weakness in the abdominal wall [6,7,8,9,10]. Thus, ALIF has been recognized as a safe and effective technique, offering several advantages over alternative methods. The surgical outcomes of ALIF highlight its efficacy [9,11,12].

The literature on return to work after spine surgery is highly heterogeneous. It generally consists of retrospective studies that include a variety of spinal pathologies and provide very different figures regarding return-to-work rates and timeframes. Furthermore, studies that include different techniques can report very different outcomes. The time to return to work can vary widely. Some studies report return to work within weeks or months, while others indicate longer periods. Patient factors such as age, gender, preoperative function, occupation, and mental health play significant roles in return to work outcomes and can lead to considerable differences in results [13,14,15,16,17,18].

With this study, we aim to contribute further insights into this field by thoroughly examining return to work, quality of life, and physical activity following ALIF surgery. Our goal is to enhance the understanding of postoperative recovery and the long-term impacts of ALIF on patients’ lives. The findings are intended to improve clinical practice and provide patients with specific recommendations to optimize their rehabilitation and quality of life.

## 2. Materials and Methods

This study received approval from our institutional ethics review board. In addition, all research was performed in accordance with relevant guidelines/regulations. Informed consent was obtained from all participants. We retrospectively identified adult patients who underwent ALIF surgery between June 2015 and June 2021 at two medical centers.

The inclusion criteria were as follows:

- Age: 18–65 years.
- Spine pathology: degenerative disc disease, trauma, infection.
- Affected levels: 1 and 2 levels.
- At least 1 year follow-up.
- Patients with no significant mental impairments that could interfere with their ability to participate in the study and participate in a postoperative telephone interview were included.

**Figure 1:**
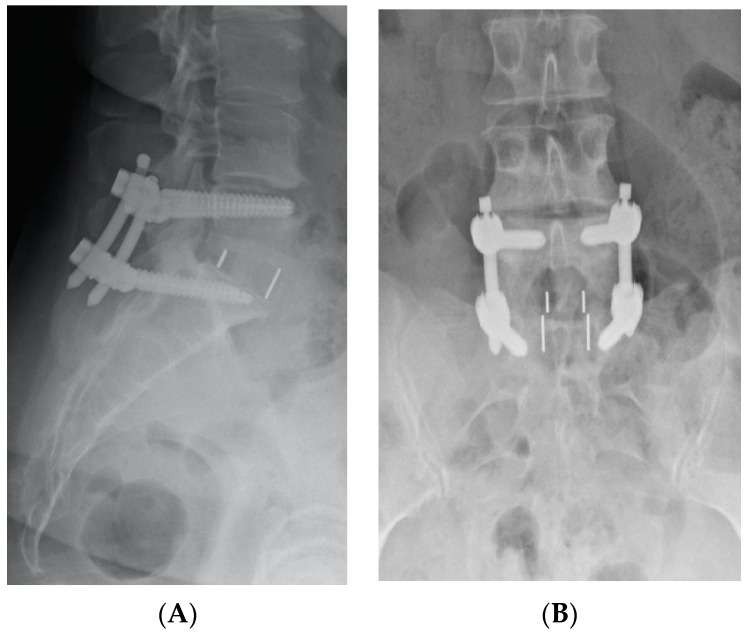
X-ray images: (**A**) lateral view and (**B**) coronal view of an ALIF procedure with a cage in the L5/S1 segment and dorsal fixation with pedicle screws.

Patients were categorized into two groups: Group A included patients who were working at the time of surgery despite experiencing back and/or leg pain, while Group B comprised individuals of working age who were not working due to their symptoms, either on sick leave or receiving an occupational disability pension. Individuals over 65 years old and retired were excluded. The surgical procedure involved an anterior retroperitoneal approach to the intervertebral disc, discectomy, insertion of a lordotic cage, and percutaneous instrumentation with pedicle screws (Figure 1).

Operative variables, such as the duration of the procedure and the number of instrumented levels, were recorded. Risk factors and pre-existing conditions assessed included nicotine and alcohol use, BMI, arterial hypertension, pulmonary disease, coronary artery disease, diabetes mellitus, underlying oncological disease, and previous musculoskeletal surgeries (hip and knee replacements). Documented complications included postoperative bleeding or hernias. Variables were selected for final analysis based on their clinical relevance and potential association with return to work (RTW). The rationale for including these variables was to capture a comprehensive picture of factors influencing RTW outcomes and identify significant predictors. Additionally, pre- and postoperative lumbar lordosis (L1-S1) and segmental lordosis in the operated segment were measured using X-rays.

The primary outcomes were the rate of patients returning to work and the number of days post-surgery until work resumption, with RTW dates self-reported in follow-up questionnaires. Details on part-time or full-time status and work restrictions were not collected. The number of patients resuming exercise activities was also noted.

For the final analysis, a standardized telephone interview was conducted to assess the clinical outcomes. To ensure standardization, the following measures were implemented:

- A detailed interview guide with scripted questions was developed to ensure all participants were asked the same questions in the same order. This minimized interviewer bias and ensured comprehensive coverage of relevant topics.
- Uniform instructions were provided to all participants at the beginning of the interview, explaining the purpose, duration, and guidelines. Interviewers were trained to avoid leading questions to prevent bias.
- A standardized form was used for recording responses, ensuring consistent data capture.
- At the end of the questionnaire, patients could answer free-text questions and add comments on difficulties encountered during their postoperative recovery.

The current condition was evaluated using the Oswestry Disability Index (ODI), the European Quality of Life 5 Dimensions 3-Level Version (EQ-5D 3L) questionnaire, and the EuroQol visual analogue scale (EQ-VAS). Patients rated their current health state on a visual analogue scale from 0 to 100, with endpoints ranging from “worst imaginable health state” to “best imaginable health state” [19]. In addition to the EQ-VAS, the EQ-5D includes five dimensions with one item per dimension: mobility (MO), self-care (SC), usual activities (UAs), pain/discomfort (PD), and anxiety/depression (AD) [20]. Current pain perception was examined using the numerical rating scale (NRS) for back pain and leg pain. A survey was also conducted regarding occupational and sports activities after surgery, as well as subjective patient satisfaction after surgery. All data were compiled in Microsoft Excel and subsequently analyzed with the statistical software SPSS^®^ (version 27).

### Statistical Analysis

Statistical analysis was conducted using SPSS (version 27, IBM, Armonk, NY, USA), with statistical significance set at *p* < 0.05. To compare differences between groups (e.g., RTW vs. no RTW), the following statistical tests were used:

Fisher’s exact test: The test was used for categorical variables to compare significant differences between groups. This test was chosen because it is more accurate for small sample sizes and when the expected frequency of some categories is low.

Nonparametric tests included the following:

- Mann–Whitney U: The test was used for comparing two independent groups on ordinal or non-normally distributed continuous variables. This nonparametric test was selected as it does not assume a normal distribution of data.
- Kruskal–Wallis tests: These were employed for data that did not follow a normal distribution.
- The Shapiro–Wilk test: This test was conducted to test the normality of data distributions. Nonparametric tests were employed where data deviated from normality, as these tests do not rely on assumptions of normal distribution.

## 3. Results

### 3.1. Patients Enrollment

**Figure 2:**
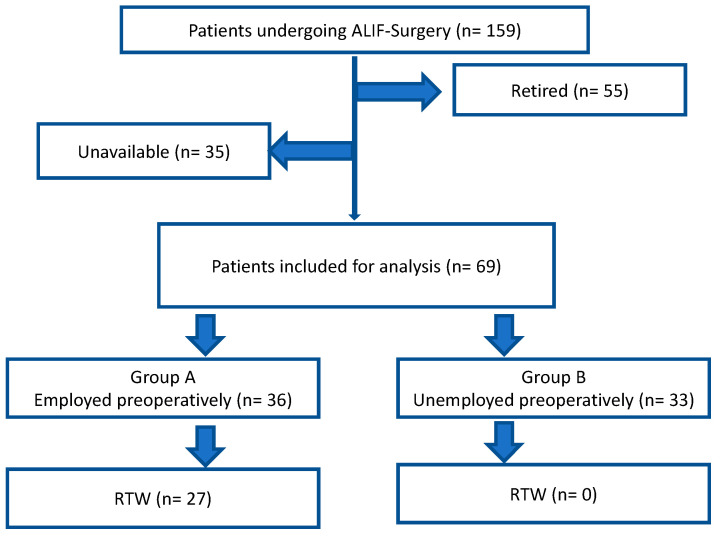
Flow diagram of included patients.

During the study period, 159 patients underwent ALIF fusion, with 55 (35%) of these patients retired at the time of surgery. Among the remaining 104 patients, 35 (34%) could not be reached for follow-up, leaving 69 (43%) patients eligible for the study. Of these, 33 (21%) were of working age but unemployed at the time of surgery, while 36 (23%) were employed (Figure 2).

Exclusion criteria: retirement at the time of surgery; patients who could not be contacted or refused to participate.

### 3.2. Return to Work

Out of the 36 patients who were employed before undergoing surgery (Group A), 9 did not return to work. The other 27 patients resumed work within a median of 3 months post-surgery. None of the patients of working age who were unemployed before surgery (Group B) returned to work post-surgery, with a significance level of *p* = 0.001. A comparison between patients who returned to work and those who did not revealed that tobacco use and previous musculoskeletal surgeries were significant predictors of failure to return to work (RTW). There were no significant differences in age, sex, history of spine surgeries, other comorbidities, or physical activity levels between the two groups. Although not statistically significant, obesity rates were higher among those who did not return to work. Additionally, patients who failed to return to work were less physically active compared to those who returned to work (Table 1).

**Figure 3:**
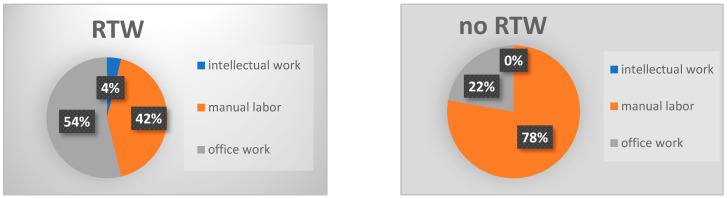
The figure shows the types of occupations, divided into different categories. No significant difference can be observed; however, in the no-RTW group, there are fewer office workers but more manual laborers.

**Figure 4:**
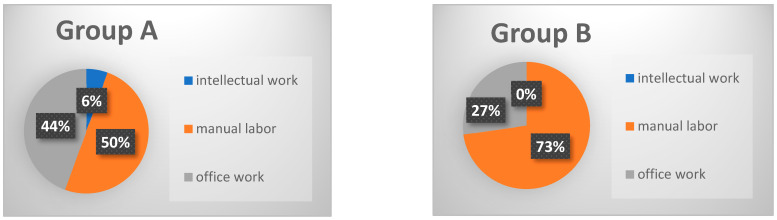
The figure shows the types of occupations in Groups A and B. No significant difference can be observed either. Nevertheless, in Group B, a lower proportion of office work and a higher proportion of manual labor could be detected.

Comparing Group A with Group B, both age and the number of comorbidities, such as coronary heart disease, arterial hypertension, and previous surgeries, were significantly higher in Group B. Manual labor was the predominant occupation in both groups, with occupational disability pensions being significantly more common among Group B patients (Figure 3 and Figure 4).

Data regarding the indication for surgery, number of fused levels, surgery duration, complications, and pre- and postoperative segmental and lumbar lordosis did not significantly impact the likelihood of returning to work after ALIF surgery (Table 2).

The median levels of leg and back pain were significantly worse in patients who did not return to work, as were the median postoperative ODI scores and EQ-VAS scores in the no-RTW group compared to the RTW group. Patients unable to return to work reported significant limitations in mobility, daily activity, and pain/discomfort compared to those who did return to work (Table 3).

There were significant differences in ODI scores, back pain, and median EQ-VAS scores in Group B. However, self-reported satisfaction after surgery showed no significant differences between the groups, with only 15% of Group B patients reporting dissatisfaction. Patients in Group B, except for the domains of pain/discomfort and anxiety/stress, exhibited severe disability in all EQ-5D 3L domains (Table 3). Postoperative rehabilitation was offered to all patients, with nearly all undergoing rehabilitation.

## 4. Discussion

The objective of this study was to delineate the patterns of return to work (RTW) following elective ALIF procedures in a large multicenter cohort. Postoperatively, 75% of all patients returned to work within a median timeframe of three months. Notably, none of the patients who were unemployed or receiving a disability pension prior to surgery (Group B) returned to work post-surgery. To understand these patterns more clearly, we analyzed patients who were employed separately from those who were unemployed before surgery.

The significance of employment in our society is profound, serving as a cornerstone of economic and social life. A 2015 survey among the German population aged between 18 and 60 revealed that employment is considered a crucial aspect of quality of life, second only to family and partnerships [21]. Moreover, employment is recognized as a source of social interaction, self-esteem, physical and mental health, and a sense of self-worth [22]. Hence, assessing levels of disability and work capability is essential for gauging the success of treatment modalities. Numerous studies have aimed to describe functional outcomes and the resumption of work across various conditions, including spine tumors, post-spine surgery, and chronic pain scenarios [23,24,25].

Previous studies have shown that the rate of RTW varies by the type of surgical intervention. For instance, discectomy and laminectomy procedures have been associated with favorable RTW rates, whereas fusion operations generally report lower rates. Singh et al. reported RTW rates of 77% for discectomy, 76% for laminectomy, and 62% for fusion [17]. Crandall et al. noted higher RTW rates: 92% for discectomy, 89% for laminectomy, and 89% for fusion [26].

The results of our study show similar outcomes, although different study designs were used for comparison. While Singh et al. compiled a cohort with various pathologies and surgical methods to examine RTW, Crandall et al. specifically focused on the role of revision surgery. This highlights the difficulty of comparing existing studies. However, specific data on RTW rates following ALIF are scarce, and distinctions between anterior and posterior fusion are rarely made. Our study attempts to address this gap, indicating positive outcomes in terms of RTW when compared to the existing literature.

Another significant concern with current and previous studies is the lack of information regarding surgeon characteristics and postoperative protocols. It is often unclear who makes the decision that a patient is unfit to work before surgery or who determines when a patient is ready to return to work. This lack of clarity complicates the interpretation of RTW outcomes, as these decisions play a crucial role in influencing recovery timelines and overall study results.

There is ongoing scientific interest in identifying predictors that either facilitate RTW or hinder and delay it. Several patient-related and treatment-specific factors have been described in this context. Age, gender, unemployment before surgery, physically demanding work, education level, psychosocial factors, and the type of surgery all play important roles in determining RTW outcomes [13,16,17,27].

In line with these reports, we identified similar factors. Important predictors for failure to RTW were smoking and previous surgeries. Although not statistically significant, an increase was observed in the number of patients who did not return to work, correlating with a higher number of comorbidities, lower physical activity levels, and being female. These factors were also confirmed in our study and could suggest potential statistical significance in larger cohorts. This underscores the importance of weight optimization and physical activity for rapid recovery and RTW. Additionally, studies have linked BMI scores with health-related quality of life (HRQL), highlighting the importance of these factors in preoperative counseling and decision-making [23,28,29,30].

An important aspect that did not emerge as significant in our study is the psychological component. Mental health factors, such as anxiety and depression, appear to influence RTW outcomes, in contrast to our findings. Studies investigating the independent effects of depression on RTW revealed that patients with a clinical diagnosis and those with higher depression scores had lower odds of RTW relative to those without depression or with lower scores in adjusted analyses [31,32,33]. This suggests that depression, whether clinically diagnosed or indicated by higher depression scores, is associated with a reduced likelihood of returning to work.

Preoperative measures such as nicotine cessation, weight loss, and initiating physical activity not only contribute to better postoperative outcomes but also facilitate RTW. A recently published review on prehabilitation, analyzing the effects of high-intensity workouts compared to standard care before surgery, showed that short, high-intensity exercise sessions can improve cardiorespiratory fitness and reduce complication risks [34]. More specifically, in the field of spinal pathophysiology, studies have demonstrated the role of muscular health, rehabilitation, and the management of associated pain syndromes in improving return-to-work rates and overall recovery [35,36,37].

Nonetheless, we do not want to overemphasize the role of physical activity. While a healthy and active lifestyle can certainly have a positive impact on recovery, it is the combination of physical strength and psychosocial factors that is crucial for achieving a good outcome.

Preoperative occupation also plays an important role in return to work (RTW) post-fusion surgery. Manual labor is identified as a potential risk factor for not returning to work, with a noted tendency towards post-surgery unemployment, although this did not reach statistical significance (Figure 3 and Figure 4). This observation aligns with previously published data analyzing work status post-surgery [17,27].

The results presented have important implications for both healthcare providers and patients. Essential for a successful recovery after surgery and reintegration into the workforce are measures that target patient behavior. Smoking cessation and, where possible, physical activity can positively influence the course of the condition. Equally important, though not directly evident from our results, are psychosocial comorbidities, which must be considered prognostic factors. It is worth emphasizing that patients who were unemployed before surgery generally show poorer outcomes, lower quality of life, and seem to have limited benefit from ALIF surgery, especially if the main treatment goal is RTW.

Given these challenges, developing an adequate approach to addressing RTW outcomes proves to be very complex. Standardization of processes is difficult to achieve, and external factors, such as the social system, cannot be controlled. Nevertheless, prospective multicenter studies should aim to establish a more comprehensive approach in a larger patient cohort to address this question.

### Limitations

This study is constrained by its retrospective nature, relying on data collected from patients during post-surgery follow-up periods, without access to preoperative scores. Consequently, our findings might be influenced by selection bias, given that only 69 out of 159 patients were included in the final analysis. This study did not explore potential strategies to enhance participation in physical exercise, nor did it assess the intensity and duration of various activities, which could provide further insights into patient recovery and outcomes. A significant limitation also stems from the characteristics of Germany’s social compensation system. The minimal difference between social compensation benefits and earnings in the low-wage sector might diminish the incentive for individuals to return to work in these areas. This hypothesis is supported by the observation that 85% of patients who were unemployed before surgery reported positive surgical outcomes but chose not to re-enter the workforce. This assumption is also supported by a Norwegian study, which investigated associations between being a disability pension applicant prior to surgery, possible confounders at baseline, and RTW 12 months after surgery. The authors found that disability pension applicants may lack motivation and incentives to return to work. Furthermore, they reported less health improvement after surgery compared to non-applicants [38]. This highlights a complex interplay between socioeconomic factors and the decision to return to work post-surgery, suggesting that financial and social incentives, or the lack thereof, play a crucial role in this process.

## 5. Conclusions

In conclusion, our study on RTW after ALIF reveals that 75% of patients resumed work within three months, although the study was limited by its retrospective nature and selection bias, with only 43% of the study cohort analyzed. The absence of preoperative data and detailed post-surgery activity levels, along with socioeconomic influences from Germany’s social compensation system, suggest a complex interplay affecting RTW motivation. These findings highlight the importance of considering socioeconomic factors and suggest a multidisciplinary approach to optimize RTW outcomes post-ALIF.

## Figures and Tables

**Table 1:** Cohort characteristics, stratified by RTW status and employment status.

Group A						
	RTW	NO RTW	*p* < 0.05	Group A	Group B	*p* < 0.05
N	27	9		36	33	0.0001
Median age in yrs	44	51.5	0.4	50	60	0.0001
Sex, female, no. (%)	16 (59)	7 (77.8)	0.4	23 (63.9)	17 (51.5)	0.3
Previous spine surgery, no. (%)	13 (48)	5 (55.5)	0.9	14 (47.2)	19 (57.6)	0.5
Smoking, no. (%)	10 (37)	7 (77.8)	0.05	17 (47.2)	13 (39.4)	0.6
Coronary heart disease, no. (%)	0	0		0	5 (15.2)	0.02
Arterial hypertension, no. (%)	7 (26)	3 (33.3)	0.9	10 (27.8)	22 (66.7)	0.0007
Obesity, no. (%)	7 (26)	5 (55.5)	0.2	12 (33.3)	16 (48.5)	0.2
Diabetes mellitus, no. (%)	3 (11)	1 (11.1)	0.9	4 (11.1)	5 (15.2)	0.7
Immunosuppression, no. (%)	0	0		0	3 (9.1)	0.1
Alcohol abuse, no. (%)	4 (15)	1 (11.1)	0.9	5 (13.9)	2 (6.1)	0.4
Cancer, no. (%)	0	0		0	3 (9.1)	0.1
Operations on the musculoskeletal system, no. (%)	1 (4)	3 (33.3)	0.04	4 (11.1)	11 (33.3)	0.04
Pulmonary disease, no. (%)	2 (7)	1 (11.1)	1.0	3 (8.3)	7 (21.2)	0.2
Sports activity, no. (%)	17 (63)	4 (44.4)	0.4	21 (58.3)	14 (42.4)	0.3
Postoperative rehabilitation (%)	26 (96)	9 (100)	1.0	35 (97)	33 (100)	1.0
Disability pension				0	27 (81.8)	0.0001

**Table 2:** Surgical and radiological data.

Group A						
	RTW	NO RTW	*p*-Value	Group A	Group B	*p*-Value
N	27	9		36	33	
Indication for surgery						
Degenerative spine disease, no. (%)	20 (74)	9 (100)	0.1	29 (80.6)	30 (90.9)	0.3
Infection, no. (%)	6 (22)	0	0.3	6 (16.7)	3 (9.1)	0.5
Trauma, no. (%)	1 (4)	0	1.0	1 (2.8)	1 (3.0)	
>1 Segment fused, no. (%)	6 (22)	3 (33.3)	0.6	9 (25)	13 (39.4)	0.3
Median duration of surgery in min.	137	152	0.5	137	165	0.4
Median preoperative segmental lordosis (°)	17	21	0.2	18	18.6	0.2
Median postoperative segmental lordosis (°)	23	26	0.5	25	24.4	0.1
Median preoperative L1-S1 LL (°)	52	46	0.6	49	47.9	0.06
Median postoperative L1-S1 LL (°)	52	48	0.9	50.4	49	0.4

**Table 3:** Outcome data.

Group A							
EQ-5D-3L	Problem	RTW	NO RTW	*p*-Value	Group A	Group B	*p*-Value
Mobility, no. (%)	No problem	15 (65)	0	0.009	15 (41.7)	4 (12.1)	0.01
	Moderate problem	9 (33)	5 (55.5)		14 (38.9)	17 (51.5)	
	Extreme problem	3 (11)	4 (44.4)		7 (17.4)	12 (36.4)	
Self-care, no. (%)	No problem	26 (96)	8 (88.9)	0.7	34 (34.4)	22 (66.7)	0.04
	Moderate problem	1 (4)	1 (11.1)		2 (5.6)	9 (27.3)	
	Extreme problem	0	0		0	2 (6.1)	
Usual activity, no. (%)	No problem	20 (74)	0	0.002	20 (55.6)	10 (30.3)	0.05
	Moderate problem	4 (15)	6 (66.6)		10 (27.8)	15 45.5)	
	Extreme problem	3 (11)	3 (33.3)		6 (16.7)	8 (24.2)	
Pain/discomfort, no. (%)	No problem	10 (37)	0	0.03	10 (27.8)	4 (12.1)	0.3
	Moderate problem	13 (48)	4 (44.4)		17 (47.2)	20 (60.6)	
	Extreme problem	4 (15)	5 (55.5)		9 (25)	9 (27.3)	
Anxiety/stress, no. (%)	No problem	21 (78)	4 (44.4)	0.2	25 (69.4)	17 51.5)	0.1
	Moderate problem	3 (11)	5 (55.5)		8 (22.3)	10 (30.3)	
	Extreme problem	3 (11)	0		3 (8.3)	6 (18.2)	
EQ VAS		80	55	0.002	70	50	0.02
ODI		12	50	0.0006	22	36	0.009
NRS back pain		3	6.5	0.002	3	5	0.006
NRS leg pain		0	6.5	0.003	2	3	0.1
Self-reported satisfaction		25 (92.5)	6 (66.6)	0.009	31 (86.1)	28 (84.8)	1.0

## Data Availability

The data presented in this study are available on request from the corresponding author.
